# Supplementary material for: Characterizing the referral care continuum among complex obstetric patients in the Blantyre District of Malawi: A mixed methods study
Source: PLOS Glob Public Health. 2025 Jul 24;5(7):e0004939. doi: 10.1371/journal.pgph.0004939 (PMC12289018; doi:10.1371/journal.pgph.0004939)
Supplement: S1 Document — (PDF) [file pgph.0004939.s001.pdf]

BLANTYRE DHO REFERRAL ANALYSIS DATA COLLECTION FORM

Name of referring facility:	Patient Name/ ID:	Patient type: Mother Neonate

Referral period: Antepartum Intrapartum Postpartum Post discharge

Referral timeline

Mode of transportation: Ambulance Personal vehicle Hired vehicle

Time complication identified:	Time transport called:	Time transport arrived:
Time of arrival at hospital:	Time first seen by provider:	Clinical escort present: Yes No

Vitals signs on arrival: BP: Pulse: Resp: Temp:	Condition on arrival: Stable Critical Dead

Pre referral diagnosis:	Admission diagnosis:

Pre referral care (tick all that were done)

Procedures:

☐ Catheterization ☐ Cannulation ☐ MRP ☐ BMC ☐ Tears Sutured ☐ Vacuum extraction ☐ Breech manouvres

☐ Knee-chest position ☐ Left- lateral position ☐ NASG ☐ Bag and mask ☐ CPR ☐ KMC ☐ Other ___________

IV Fluids:

☐ Normal Saline/ Ringer's lactate ☐ Dextrose 5%/ 50% ☐ Haemacele ☐ Other _________________________

Medications:

☐ Anticonvulsants ☐ Antihypertensives ☐ Antibiotics ☐ Uterotonics ☐ Tocolytics ☐ Corticosteroids

☐ Analgesics ☐ Laxatives ☐ Other ____

Referral outcome: Discharged Died

Date of referral: **Date of outcome:** **Time of outcome (if on same day as referral):**

Form completed by: __________________________ **Designation** ____________________________

Signature___________________________________ **Date:** __________________________________
