# Supplementary material for: Characterizing the referral care continuum among complex obstetric patients in the Blantyre District of Malawi: A mixed methods study
Source: PLOS Glob Public Health. 2025 Jul 24;5(7):e0004939. doi: 10.1371/journal.pgph.0004939 (PMC12289018; doi:10.1371/journal.pgph.0004939)
Supplement: S2 Document — (PDF) [file pgph.0004939.s002.pdf]

DATA COLLECTION TOOLS (ENGLISH)

a) Focus Group & In-Depth Interview Guide (For Clinicians and Nurse Midwives)
Main Study Title: Improving the referral process for obstetric emergencies: a midwifery-led project in Blantyre District, Malawi

Date of focus group ___________________________<br><br>Location of focus group________________________<br><br>Cadre of participant(s)<br><br>_____________________ _____________________<br>_____________________ _____________________<br>_____________________ _____________________<br>_____________________<br><br>Length of discussion ________________<br><br>Initials of facilitator _________________ Initials of recorder _______________________
Materials Needed:<br><br>• 1 recorder<br>• 2 copies of consent form for each participant<br>• A copy of this guide<br>• Pens for note taking and participant consent signing

A. ***Introduction Process (10 minutes)***

1. Welcome participants as they arrive at the focus group venue. Get them seated around a table. The researcher should also sit around the same table. If there is a note taker, they should sit outside the table so that her note taking does not disturb the group dynamics.

2. The researcher and note taker introduce themselves.

3. Ask the participants to introduce themselves by saying their first name only

4. Explain the purpose of the focus group session by saying:

"This group/individual [circle one] discussion is meant to learn about your perspectives towards the referral process to a tertiary health facility.

This session will not be used to review your job performance as a nurse, it will only be used to inform GAIN about how we can support the work of nurses to improve maternal and neonatal child care. You do not have to participate in this session. Choosing not to participate in this discussion will not affect your training and mentorship with the GAIN project (if Nurse-Midwife).

The discussion will remain confidential and I ask you all to uphold this confidentiality when leaving the room. Your opinions will be transcribed anonymously and they will be used by the research team and for improvement purposes only. If you agree, I will tape this discussion as it will help me to recall what has been said and to prepare a transcript of the session. But, despite being taped, I would like to assure you that the discussion will remain anonymous. The tapes will be kept safely in a password protected device until they are transcribed word for word, then they will be destroyed. The transcribed notes of the meeting will contain no information that would allow you to be linked to specific statements. If there are any objections to this, please let me know now."

B. ***Discussion Period (30+ minutes)***

Note for interviewer: The following topics and questions are suggestions to help direct the discussions. Please note that you can allow the topics to evolve naturally and that you may not get to every point listed below.

Midwives (Health Centre)

1. Tell me about your experience in referring clients. Can you tell me a recent referral story?
2. What are the protocols for referral?
3. Based on your experiences, how do your patients perceive being referred?
4. What do you need to do your job better in the referral process?

Midwives and Clinicians (QECH)

1. Tell me about your experience in receiving referred clients. Can you tell me a recent story about a referral client you received?
    a. What went well?
    b. What did not go well or could be improved?
2. Based on your experience, how do patients perceive being referred?
3. What do you need to do your job better in the referral process?

b) Focus Group & In-Depth Interview Guide (For clients)
Main Study Title: Improving the referral process for obstetric emergencies: a midwifery-led project in Blantyre District, Malawi

<table>
<tr><td>Date of focus group ________________________

Location of focus group_____________________

Age and parity of participant(s)

____________________ ____________________
____________________ ____________________
____________________ ____________________
____

Length of discussion ______________

Initials of facilitator _______________ Initials of recorder ____________________</td></tr>
<tr><td>Materials Needed:

• 1 recorder
• 2 copies of consent form for each participant
• A copy of this guide
• Pens for note taking and participant consent signing</td></tr>
<tr><td></td></tr>
</table>

A. Introduction Process (10 minutes)

1. Welcome participants as they arrive at the focus group venue. Get them seated around a table. The researcher should also sit around the same table. If there is a note taker, they should sit outside the table so that her note taking does not disturb the group dynamics.

2. The researcher and note taker introduce themselves.

3. Ask the participants to introduce themselves by saying their first name only

4. Explain the purpose of the focus group session by saying:

“This group/individual [circle one] discussion is meant to learn about your perspectives towards the referral process to a tertiary health facility.

This session will only be used to inform GAIN about how we can support the work of nurses to improve maternal and neonatal care. You do not have to participate in this session. Choosing not to participate in this discussion will not affect your reception of postnatal or any other health care services.

The discussion will remain confidential and I ask you all to uphold this confidentiality when leaving the room. Your opinions will be transcribed anonymously and they will be used by the research team and for improvement purposes only. If you agree, I will tape this discussion as it will help me to recall what has been said and to prepare a transcript of the session. But, despite being taped, I would like to assure you that the discussion will remain anonymous. The tapes will be kept safely in a password protected device until they are transcribed word for word, then they will be destroyed. The transcribed notes of the meeting will contain no information that would allow you to be linked to specific statements. If there are any objections to this, please let me know now.”

Age:	
Referred from:<br><br>Referred to:	Referred during :<br><br>☐ Antenatal<br>☐ Intrapartum<br>☐ Postnatal
Number of pregnancies:	
Number of births:	
Primary diagnosis:	☐ Hemorrhage (APH/ PPH)<br>☐ Pre-eclampsia/eclampsia<br>☐ Prolonged labor<br>☐ Obstructed labor<br>☐ Preterm labor<br>☐ Sepsis<br>☐ Big fundus

	☐ Young/ Short Primgravida<br>☐ Grand multigravida<br>☐ Underlying medical condition (specify) ___________________<br><br>☐ Past history of obstetric complications (specify) ____________________<br>☐ Other: ______________________<br>☐ Does not know

B. <u>Discussion Period (30+ minutes)</u>

Note for interviewer: The following topics and questions are suggestions to help direct the discussions. Please note that you can allow the topics to evolve naturally and that you may not get to every point listed below.

<u>Clients:</u>

1. Can you please tell me about the referral process for your birth?
   a. What was the reason for your referral? (confirm diagnosis)
   b. Was the referral done during antepartum, intra or postpartum?
   c. Which health facility were you referred to?
   d. How did you feel about being referred?

2. Can you tell me about how you got from the HC to the hospital?
   a. Was the referral process timely or delayed?
      i. If there was a delay, can you tell me a little more about the delay?

b. What were the financial costs incurred for transport (if any)?

3. What do you think are the most common reasons for referral?

4. In your opinion, what can be done to enhance the referral process for women who may need to go to another facility?
