# Supplementary material for: Characterizing the referral care continuum among complex obstetric patients in the Blantyre District of Malawi: A mixed methods study
Source: PLOS Glob Public Health. 2025 Jul 24;5(7):e0004939. doi: 10.1371/journal.pgph.0004939 (PMC12289018; doi:10.1371/journal.pgph.0004939)
Supplement: S1 Table — (PDF) [file pgph.0004939.s003.pdf]

Table of Referral Interval (Hours) by Pre-referral Diagnoses

Pre-referral Diagnosis		Referral Interval in Hours: Median [Range]	Referral Interval in Hours: Mean (SD)	p-value
**Antepartum Hemorrhage**	Yes (n=14)	1.58 [0.500, 14.2]	3.28 (4.55)	0.438
	No (n=384)	2.00 [0.417, 29.7]	3.09 (3.99)	
**Fetal Distress**	Yes (n=35)	1.73 [0.450, 14.7]	2.31 (2.80)	0.121
	No (n=363)	2.00 [0.417, 29.7]	3.17 (4.11)	
**Postpartum Hemorrhage**	Yes (n=19)	2.42 [1.67, 5.17]	2.66 (1.07)	0.148
	No (n=379)	2.00 [0.417, 29.7]	3.12 (4.12)	
**Pre/eclampsia**	Yes (n=80)	2.15 [0.717, 13.8]	3.02 (2.43)	0.138
	No (n=318)	2.00 [0.417, 29.7]	3.11 (4.30)	
**Premature labor**	Yes (n=13)	2.00 [0.750, 16.1]	4.56 (5.32)	0.427
	No (n=385)	2.00 [0.417, 29.7]	3.05 (3.96)	
**Retained placenta**	Yes (n=12)	1.33 [0.583, 25.5]	4.92 (8.47)	0.534
	No (n=386)	2.00 [0.417, 29.7]	3.03 (3.78)	
**Prolonged/ Obstructed labor**	Yes (n=129)	1.90 [0.417, 27.0]	2.30 (2.83)	0.031
	No (n=269)	2.08 [0.450, 29.7]	3.56 (4.50)	
**Other maternal diagnoses**	Yes (n=116)	2.03 [0.500, 29.7]	3.99 (5.48)	0.518
	No (n=282)	2.00 [0.417, 27.0]	2.80 (3.37)	
