# Supplementary material for: Characterizing the referral care continuum among complex obstetric patients in the Blantyre District of Malawi: A mixed methods study
Source: PLOS Glob Public Health. 2025 Jul 24;5(7):e0004939. doi: 10.1371/journal.pgph.0004939 (PMC12289018; doi:10.1371/journal.pgph.0004939)
Supplement: Attachment — Submitted filename: Response to Reviewers.docx [file pgph.0004939.s006.docx]

Hannah Tappis, DrPH, MPH
Academic Editor
PLOS Global Public Health

Re: *Characterizing the referral care continuum among complex obstetric patients in the Blantyre District of Malawi: A mixed methods study* (PGPH-D-24-00015)

Dear Dr. Tappis, Reviewer 1, and Reviewer 2,

Grateful for the opportunity to revise, please accept this resubmission of our Manuscript and Revised Manuscript with Track Changes. The thoughtful comments and ideas from both reviewers challenged our team to revisit a variety of components and ultimately improved our final product. We are glad to provide a **Point-by-Point Response to Reviewer Comments** in the attached table. We particularly appreciated that the suggested revisions improved the specificity of reporting across our methods and results which we believe further highlights the key findings.

In addition to the revisions described below, you will find that we have replaced all specific clinical names with pseudonyms such as “Facility A”, “Facility B”, and so on. Since our initial submission in January, our team implemented a policy that we would anonymize clinical sites in publications of this nature in order to preserve their privacy. Along with emailing about this edit on October 9, 2024, we have made an additional note of this within our revised methods section to clarify this.

We would be happy to further discuss any of our responses in the attached table or the corresponding edits in our Manuscript. We trust you will find this resubmission to be an improved product that would be of strong interest to interdisciplinary readers with interest in global public health, particularly in Malawi and similar settings. We look forward to hearing from you again soon.

Warmly,

Oveka Jana Mwanza

| **Point-by-Point Response to Reviewer Comments** *Reviewer 1: Comments 1 through 37*  *Reviewer 2: Comments 38 through 48* | | |
| --- | --- | --- |
| **Num** | **Comment** | **Response** |
| 1 | The authors provide an informative introduction to the study including key maternal health statistics at national and subnational level. The authors also highlight some elements related to referral care including issues arising from pre-referral care. However, a few elements seem inadequately addressed in the introduction, which the authors should consider clarifying. 1) referral care continuum? This is a key concept in their title but not very well explained in the introduction? What is the scope? Is it a hierarchy? Etc. 2) complex obstetric patients? A sentence or two clarifying this, in light of referral and other issues would be helpful. | We appreciate the opportunity to clarify. We have added a brief definition, and the sentences now read: "Exploration of barriers and bottlenecks in other areas across other health facilities and geographical areas could lead to similarly targeted solutions. Informed approaches would be likely to support quality referral care from the time a decision to refer is made until the patient receives needed care at the receiving facility—across the “referral care continuum”." |
| 2 | L45, suggest removing ‘antenatal’ because it should be part of “maternal health care”? | We accept this suggestion and have deleted the word "antenatal" from this sentence. |
| 3 | L47, authors should specify ‘skilled attendants’ where? Is it in Malawi or SSA? | We're happy to clarify! The sentence immediately prior specifies that this paragraph is discussing Malawi, and the end of the sentence referenced here says "in the country". Therefore, we've left this as is and feel it is specified already though the reviewer may have missed this initially. |
| 4 | Authors should improve coherency between the first and second paragraph. There is a disconnect. Perhaps rephrase L57 to be specific to the facility delivery coverage? | We have replaced "These statistics" with "High mortality rates despite high rates of skilled attendance at birth" which we believe more specifically connect the paragraphs to address this concern. |
| 5 | L60, It could be helpful for authors to specify where the pre-referral challenges were reported from? Which type and level of facilities? | We have added "at secondary and tertiary hospitals" to more specifically summarize the study described here. |
| 6 | L66, ‘exploration of barriers and bottlenecks in other areas….? Which areas? Geographical? Clinical? Referral continuum??? It would be helpful for authors to clarify. | We have replaced "other areas" with "across other health facilities and geographical areas" for increased specificity. |
| 7 | L90, perhaps the third word should be “understand” instead of understanding? | Thank you for bringing this to our attention. We have updated to "understand" for grammatical accuracy. |
| 8 | L105, the section “setting and participants” seems to only include setting? | We have updated this subheading to "Study Site" to better reflect its contents |
| 9 | L125-126, the definition for “referral interval” seems inconsistent. “…..to refer to the charts in this sub-analysis involving maternal charts which……” | Grateful for the opportunity to clarify, we have updated this section to now read "We use the term “referral interval” throughout to refer to the time between the identification of a complication prompting a referral and the time at which that patient was seen by a provider at the Hospital—this temporal period falls within the broader referral continuum concept. " We believe that by separating our definition of the referral interval from the fact we conducted a sub-analysis for this adds clarity. |
| 10 | L130, could authors specify period referred to as “pre-COVID” charts? | We have specified this in L130-133: "*To quantitatively assess pre-referral care practices, patient charts of any woman referred from one of seven referring facilities to QECH between March 2019 – March 2020 were extracted and reviewed. This baseline cutoff was chosen in part to avoid confounding in the care process involving national shutdowns during the COVID pandemic.*" |
| 11 | § Under researcher characteristics – authors state that they used the word “woman/women”… but in the methods they seem to use “patients”? is this different? | We have added "*and/or “patient/patients”*" here to clarify--we provide this statement to justify deviance from the increasingly accepted gender-neutral term of 'birthing person'. |
| 12 | Authors should clarify on how the referral interval was computed, its not very clear as is | Please see above response. We believe that the revision to more succinctly/separately define referral interval addresses this comment as well. |
| 13 | The authors provide a range of results with good interpretation. A few things to point out for authors to consider. Overall it is not very easy to follow all the results presented, and there is inconsistent use of terminologies that are also not defined by the authors. Also, there are inconsistencies in variable nomenclature across tables in the results section, that is, one seemingly similar variable labelled differently in different tables. Perhaps providing a clearer scope of the work might help refine the presentation and consistency of the results. As previously highlighted, authors do not clarify how they operationalised “referral continuum” hence the range and mix of results that currently makes it hard to appreciate. What is the focus? What are the key elements of interest? Even the qualitative results could use some refining. It would be helpful for the results to sort of show or apply to the continuum instead of the way they are now mixed. Let us appreciate the issues with pre-referral care, then go on to understand (the consequences of this) reflected by quality of care at the receiving facility?  There seems to be a number of goal-posts that are not synchronised – ‘characteristics of pre-referral care driving poor outcomes’; ‘characterise obstetric care and outcomes across referral continuum and explore facilitators and barriers to the referral process’; ‘quality of pre-referral care and health outcomes’; ‘facilitators and barriers to timely quality obstetric referrals’……Authors should harmonise these, provide a clear scope then arrange results within that scope. | We also believe that the revision to more clearly define "referral interval" and the sub-analysis we conducted adds flow to the results. To align the language, we have updated table column headings across tables to match other subheadings which specify either 1) All Maternal Referrals or 2) Maternal Referrals for whom a referral interval could be calculated. |
| 14 | L212, the term ‘intrapartum period’ has not previously been defined | For those who may not be familiar with this common term, we've updated the sentence accordingly: "Skilled attendants are primarily nurse-midwives who provide 72% of antenatal care and 68% of intrapartum care from the onset of labor through the delivery of the placenta (“intrapartum care”) in the country." |
| 15 | L215, the Fisher’s exact tests revealed no significant differences in what? | This full sentence now reads: "*Fisher’s Exact Tests revealed no significant differences between the maternal referral characteristic variables of the full sample of pre-COVID maternal referrals and the subset for whom referral interval could be calculated used in the sub-analyses.*" We believe this more clearly states there weren't differences between the full sample and the sub-analysis sample related to the Table 1 characteristics. |
| 16 | Authors should consider revising title of Table 1 | We have updated it to "Maternal Referral Characteristics for the Full Sample and the Sample use for Sub-Analysis" to align with the language updates described in the above response throughout the Results section. |
| 17 | Table 1, the variable ‘facility’, should it be ‘referring facility’ with another word to speak to the data there i.e., distance? | We have updated this variable to "Referring Facility by Distance from QECH" for added clarity |
| 18 | A number of terms or variables in Table 1 have not been defined | From our understanding, the only variable that may not be familiar to the average reader would be 'pre-referral conditions' which we explain prior to presenting the table in L239-243: "Most women presented one pre-referral condition (90.45%)—most commonly prolonged/obstructed labor followed by pre/eclampsia and other diagnoses." We are unclear as to which terms this Reviewer is referring to. Based on prior comments perhaps it is referral period, which are common terms (see above). |
| 19 | What is other ward in table 1? | "Other ward" here refers to any clinical ward that was not the labor or antenatal ward; a note to clarify this has been added to the bottom of the table |
| 20 | L220, what is meant by “referral characteristic”? | We have added "*aspects of a patient’s health or care during the referral period*" to more clearly define this. |
| 21 | Table 2, authors should clarify which statistic the p-value refers to | While this comment isn't entirely clear to us, we wish to clarify that we have utilized p-value as typically used in statistical analysis. As noted in the Methods section Kruskal Wallis and Fisher's exact tests were used to analyze. We overserved that according to journal publishing practices it would be unusual to state specific tests upon each use in the results. Accordingly, we've left Table 2 p-values as is. |
| 22 | L234, it would be helpful for authors to clarify reference for establishing that r=0.2 is ‘moderate correlation’? | We believe that this is implied by the sentence were we explain moderate correlation was found. We have provided the specific statistic in the case that a reader wishes to draw their own conclusions about the extent of correlation as the research is nuanced. |
| 23 | The section on the quality of data from the charts, L287-295 should come earlier, perhaps after participant characteristics. | Thanks for this observation. We agree. We have moved this to the section immediately following the summary of participant characteristics. |
| 24 | L311, I would suggest authors to expand the current quote or find an alternative quote. The present one does not seem to fit well with the point put forward on long wait times and poor quality of care | We have included a sentence clarifying that high patient to provider ratios contribute to care delays across the district. |
| 25 | L314, presuming that this text is in reference to QECH, I suggest the authors use referring facilities (for the lower-level facilities) and receiving facility (for what at the moment I think is termed as ‘referral hospital?’) to avoid confusion | As outlined in our rebuttal letter text, we have updated our language referring to QECH throughout to be consistent with "the Hospital" to avoid confusion and align with recently set group policies. |
| 26 | L339, the sentence “participants perceived an insufficient supply of ambulances in high demand” is not clear, authors should consider revising it | Thank you for the chance to clarify. We have removed "in high demand" and believe that improves the sentence overall. |
| 27 | L361, the authors should clarify in brackets, the “it” referred to in the quote | We have added "[a lack of communication]" to this quote for clarity. |
| 28 | Although consistency especially of the first two or three paragraphs could be improved. Also, generally the implications of the findings are inadequately addressed. Beyond Malawi, what should other SSA countries take from the study? But also interesting the “framework” that has been used for the discussion, which is not in methods or results. Authors should look into such inconsistencies… Why is referral interval not discussed? It seemed an important element of the study? | We are unsure about what the reviewer wishes to see in terms of "consistency especially in the first two or three paragraphs". In terms of broader implications, we believe that we addressed this throughout by starting each subheading section with statements like 'optimal referral systems' or 'ideal referral hospitals’'. As with most mixed methods and particularly qualitative research, we wish to avoid over extrapolating our findings and therefore intentionally avoided direct statements about its application to SSA countries more broadly. Our findings are indeed most relevant to Malawi as we share in the results. The reviewer also brings up a relevant point about the application of the framework in the discussion. Our approach wasn't designed this way, and it would be disingenuous to state that we had done so retrospectively. Still, we understand we need to better describe this reality and are grateful for the opportunity to do so. We have updated our Methods section (L 211-215) to better describe this and the Discussion section (starting at L 470) to align with that. |
| 29 | L395, confirmed??? | We describe this issue in our background and do believe our findings confirm this. We have cited at the end of the sentence accordingly. |
| 30 | L405-407, wondering why authors haven’t included work from other sub-Saharan African countries that have demonstrated the effect of distance and travel time with poor maternal and perinatal health outcomes? Perhaps they should also specify where the work referred to is underway? | Thanks for the opportunity to revisit this. We have incorporated a few additional sentences and references to discuss where and how our findings overlap with other work in SSA and low-resource settings in L 451-454 and L 461-465. |
| 31 | L508, “Impact of social support” – this sections seems to have a mix of social and health system elements which I suggest be separated. The part of patients not being given clear reasons etc from L514, should be put in the section on communication, it is a measure of quality of care provided by the providers/system?. then, based on the narrative of “social support”, it seems more of social influence hence authors should specify that given that social support is broader than social influence. | We have included a description that social support in this context is more than social influence, but rather the broader system of support for both patients and their support systems to thrive. We have also moved the data about lack of clear communication from provider to patient to the systems/ communication section. |
| 32 | L524, which component of care? ….this further speaks to the inconsistent use of terminologies or clear operationalisation of key constructs. Please harmonise | We added "referral" to specify type of care. |
| 33 | L526, the first recommendation – considering populations needs were not assessed in the present study, so based on challenges identified, could the authors be clear on what “especially increasing nurse-midwives” would help solve. | We believe that this is addressed within the same sentence where we state this is needed "to accommodate the growing needs of the population and to enable respectful and timely care". We make the case throughout the introduction that nurses and midwives (the providers throughout our study) provide most maternal health care. |
| 34 | L539, the link between supplies and documentation is not clear, and was not reflected in their results – the results mostly linked it to patient load? | While it is true the link warrants further study, we also present the impact of missing supplies on care overall (i.e. L 300-308) which prompted this recommendation. |
| 35 | The first and last bullets under the suggestions of L533, can’t they be merged? Its all training, so specify what training for who and by who. And the text that follows it from L543? Can’t these be integrated? | Respectfully, we disagree with this comment. Aligning with previous comments that call for specificity and clarity, we wish to keep the first bullet (specific to referring facilities) separate from the last (referral/receiving facilities). We also believe the following text provides broader context of training needs with justification from prior research. |
| 36 | The implication(s) of the first limitation does not come out clearly | We have added "which restricted the number of analyses we could perform." Which we believe better introduces the following sentence to explain this: "Unlike comparable studies which could calculate post-referral hospital wait-time separate from transport time, our referral interval spanned from the time of complication at a referring facility to the time seen by provider." |
| 37 | L584, the suggestion is not very clear | We have added "and their impact on patient outcomes" for improved specificity in this suggestion. |
| 38 | Introduction: I would make first paragraph two paragraphs. First paragraph dedicating problem statement: despite universal access to SBA, maternal and neonatal mortality is still high. Second paragraph would be dedicated to possible challenges for that. Third paragraph could be national efforts to address those challenges. Then the fourth and fifth paragraphs could be rationale and significance of the study. | Thanks for this comment. In our understanding, our first paragraph does outline the problem in this way (despite access mortality is still high). Similarly, the second paragraph outlines challenges, particularly Lines 60-71. We have added the sentence "Additionally, there is a lack of national efforts to address referral care issues in Malawi." to the end of our third paragraph, and then the final paragraphs also align with your proposition to include rationale of the study. |
| 39 | What is the theoretical foundation of the study: the Three Delays Model or the 5S Model? Clarifying this would be easier if the researchers developed a conceptual framework illustrating the relationship between outcome and predictor variables. Clearly define the outcome variable(s) of interest for the analytical part of the study (e.g., maternal condition on arrival, maternal death, and/or referral interval) and the predictor variables (e.g., facility-level and individual-level predictors). | We didn't have a preconceived theoretical foundation for the study. While this is common among qualitative research papers it is more mixed with concurrent mixed methods studies like ours. We appreciate that we needed to clarify this in our methods more explicitly and therefore added a brief summary to explain our approach and why we ultimately applied the 5 S model. Our study did draw specifically from the Three Delays Model, instead we wished to comment on the fact that our findings highlight barriers that are discussed with this common way of understanding things. Similar to our other comments about significant missingness in the data and our hesitation with multivariate modeling (described below), we don't yet feel as though we have a robust enough understanding to provide a model accurately depicting predictors. Instead we wish to present these bivariate findings of an under researched topic to share our first findings from this work and encourage additional research to build upon this. |
| 40 | It’s great that mixed methods research is being used. Please indicate where both qualitative and quantitative methods were integrated—specifically in the Results or Discussion sections. | Our approach to concurrent application of the methods is outlined in Lines 207-211 where we specify that we specifically explore triangulation in the Discussion section. |
| 41 | Have you assessed whether the sample size is adequate for subgroup analysis? | Because we found significant differences, we did not choose to retrospectively assess for power as that would be computed with a Type II error in mind. We did share our overall sample size calculation but by nature of significant results in the sub-analyses it would be unusual to calculate power. |
| 42 | Please describe how the researchers ensured the trustworthiness of the qualitative components of the study, either in the Methods section or the Discussion section. | We have added a few sentences about the steps we took to ensure the components necessary to ensure the trustworthiness of qualitative data were followed. |
| 43 | Analysis: It is unclear why multivariable regression accounting for clustering was not performed. Please conduct a multilevel regression analysis and report regression coefficients and 95% confidence intervals to identify predictors. | We also wish that we would have felt confident to perform a multivariate analysis. As we noted in our results, we were concerned with missingness in the data generally and we were only able to conduct referral-related analysis on a subsample. We've added a specific statement "Further multivariate analyses of more robust data sets would also support the identification of potential predictive factors in the presence of confounders.". |
| 44 | To improve the distribution of values in each cell, I would recategorize several variables. For instance, in Table 2, consider recategorizing the following variables: pre-referral diagnosis, diagnosis upon admission, hospital procedures, and medications given. | We understand the desire for combining categories, however our clinical partners presented the data in this way and we have proactively chosen to preserve their categorization. |
| 45 | In Table 2, the variables "distance to hospital" and "average hours to see provider" are likely to be correlated with each other. Similarly, in Table 5, the variables "facility" and "distance" may also be correlated. Please check for multicollinearity among these variables. | We checked for correlation between "distance to hospital" and "average hours to see provider" using a Spearman’s test and found a very low positive, insignificant correlation (rho:0.110, p-value 0.09); we also assessed the same for "facility" and "distance" and found these variables were positively correlated (rho: 0.682, p-value >0.001). Accordingly, we added a sentence to share this added finding. We left all variables from the referral form in the results for use by our clinical colleagues, despite not testing them against one another. |
| 46 | In Table 5, recategorize the "model of delivery" variable. Combine "forceps" and "vacuum" into a single category labeled "assisted delivery," and categorize "breech delivery" under "spontaneous vaginal delivery" or "assisted delivery" as appropriate. | We again chose to do this as it aligns with how our clinical partners assess and view data. They have shared that there are nuanced differences in delivery type in their experience, so our results follow suit. Additionally, we unfortunately do not have data on which, if any, of the breech deliveries were assisted vs. spontaneous and are therefore hesitant to lump them into an "assisted delivery" category. We have added a footnote to the table sharing this. We did run a quick bivariate test and found no difference in p-value when forceps and vacuum were combined vs. separate. |
| 47 | The qualitative findings did not clearly identify the barriers and facilitators of pre-referral practice as outlined in the study's objectives. Additionally, I would expect to see an analysis of provider behavior as either a barrier or facilitator of referral practices. | Thanks for your thoughtfulness here. Still, we disagree, as the qualitative findings identified key barriers and facilitators to pre-referral care and referral practices such as high patient to provider ratios impeding timely care, the lack of a formal inter-facility communication system, and the importance of transparency and communication between referring providers and their patients. Under the scope of this study, we could not analyze provider behavior as that would be best explored through an ethnographic approach, though it is an area we would like to explore in future studies. |
| 48 | Discussion: A bit of reorganization is needed. Summarize key finings in the first paragraph then interpret and discuss its implications in subsequent paragraphs and describe “triangulation of the findings” in the methods section. | While we appreciate that this would be one approach, we have described in our methods (see above) that we planned to discuss triangulation of the results in our discussion section. Because we applied a concurrent/parallel approach (vs. exploratory or explanatory) we believe this is a reasonable approach. |
