# Supplementary material for: Characterizing the referral care continuum among complex obstetric patients in the Blantyre District of Malawi: A mixed methods study
Source: PLOS Glob Public Health. 2025 Jul 24;5(7):e0004939. doi: 10.1371/journal.pgph.0004939 (PMC12289018; doi:10.1371/journal.pgph.0004939)
Supplement: Attachment — Submitted filename: Response to Reviewers_2.docx [file pgph.0004939.s007.docx]

Annesha Sil, Ph.D.

Staff Editor

PLOS Global Public Health

Re: *Characterizing the referral care continuum among complex obstetric patients in the Blantyre District of Malawi: A mixed methods study (PGPH-D-24-00015)*

Dear Dr. Annesha Sil and Reviewer,

Thank you for another opportunity to strengthen our manuscript. **Please find the resubmission of our Manuscript and Revised Manuscript with Tracked Changes, along with a point-by-point response to reviewer comments below.** With this re-revision we are particularly glad for the opportunity to being additional clarity and transparency to our methods. We appreciate the reviewer’s attention to detail and recognition of this work’s value.

| **Point-by-Point Response to Reviewer Comments, Part 2** | | |
| --- | --- | --- |
| **Number** | **Reviewer Comment** | **Response** |
| 1 | Line 49 pg4: the sentence ends abruptly, what solutions are referred to here? | While we were referring to improvements in maternal health broadly, upon re-reading this sentence here we have chosen to remove it we agree it wasn’t necessary as written. |
| 2 | Line 57-60 pg 4: missing punctuation | Thank you for bringing this to our attention, we have added two commas to this sentence to improve readability. |
| 3 | Lines 96-101 pg. 6: please revise the text to more clearly outline the study's primary aim and specific objectives. Additionally, we encourage you to emphasise how the study contributes to the broader scientific knowledge and clarify its potential relevance and value beyond the specific context of this location, as suggested also by previous reviewers. | We have added the following sentence to the summary of our study objectives: “In addition to informing local efforts to strengthen maternal referrals, this study aimed to contextualize the downstream health outcomes of existing care gaps and to inform solutions in settings with limited resources.” We believe this better alludes to the additional relevance we outline in the discussion section. |
| 4 | What methodological orientation underpins the qualitative aspect of the study? | We have updated lines 198-199 to clarify that our inductive coding approach aligns with grounded theory methodology. |
| 5 | Could you please clarify the rationale for using both interviews and focus groups in your study, and how participants were assigned to each method? It would be helpful to understand the specific purpose of each approach and the criteria guiding participant allocation. | Thanks for the opportunity to clarify, we have added two clarifying sentences beginning at line 177: “Recruitment for focus groups and interviews occurred during the same period on differing days—eligible participants who were available on the day each method occurred were invited. We used both in-depth interviews and focus-group discussions to enable us to capture individual experiences and communal perceptions around care respectively.” |
| 6 | Line 185-186: for transparency, please provide more detail on the specific variables included in the quantitative analysis, for example, which maternal care practices and outcomes were considered? And what was the rationale behind the selection of variables to include in the model? | We have updated lines 198-202 to further unpack the variables chosen and rationale. |
| 7 | The discussion section is currently quite lengthy (10 pages). Please streamline it to focus more directly on the key findings and their implications. | The discussion was lengthened in response to prior reviewer comments. Still, we recognize the length reduces flow. We have made cuts and edits throughout (i.e. lines 480-483, 488, 502, 516-520, 536-541, 548-550, 569, 589-590, 606-607, 659-660, 665, 682). |
| 8 | Please consider moderating the recommendations to ensure they are more closely aligned with the study's findings. At times, they appear to reflect personal opinions rather than being directly supported by the data. | We have removed the prior section outlining broad national recommendations (reflected in line 604) to more narrowly focus on the recommendations aligning with the conclusions of our study. We agree this streamlines and emphasizes more immediate solutions. |

We look forward to your review and reply. We anticipate you’ll find this resubmission to be further improved and of strong interest to your readers. We commend you for continuing to elevate global health work during this challenging time.

Cheers,

Oveka Jana Mwanza
